# Immune cells during compensatory renal hypertrophy after unilateral nephrectomy

**DOI:** 10.21203/rs.3.rs-9568023/v1

**Published:** 2026-05-15

**Authors:** Shishir Kumar Patel, Qisen Guo, Mahta Gooya, Ryo Matsuura, Hyun Jun Jung, Radhika Kapoor, Tara Fallah Rastegar, Sanjeev Noel, Hamid Rabb

**Affiliations:** Johns Hopkins University School of Medicine; Johns Hopkins University School of Medicine; Johns Hopkins University School of Medicine; Johns Hopkins University School of Medicine; Johns Hopkins University School of Medicine; Johns Hopkins University School of Medicine; Johns Hopkins University School of Medicine; Johns Hopkins University School of Medicine; Johns Hopkins University School of Medicine

**Keywords:** Compensatory renal hypertrophy (CRH), Unilateral nephrectomy (UNx), Glomerular filtration rate (GFR), Kidney mononuclear cells (KMNCs)

## Abstract

The mechanisms underlying compensatory renal hypertrophy (CRH) in living kidney donors after unilateral nephrectomy (UNx) remain complex and underexplored. We examined immune cells involved in CRH after UNx in mice. Following UNx of the left kidney in WT mice, GFR measurements, flow cytometry, and single-cell RNA sequencing were performed over 8 weeks. Rag1^−/−^ mice were studied to explore role of adaptive immune cells in GFR changes during CRH. Immune cells showed dynamic, time-dependent changes after UNx. Innate immune system responded rapidly, with increases in neutrophils and macrophages, while NK cells initially decreased. Subsequently, adaptive immune cells, including CD4^+^, CD8^+^, and DN-T cells, showed changes in resident marker CD69 and regulatory markers such as CD4^+^CD25^+^. Expression of PD-1, CTLA-4, and TIGIT on T cells changed during acute injury (0–24 hours), recovery phase (24 hours-1 week), and immune remodeling (4–8 weeks). GFR was significantly higher in later recovery phase in Rag1^−/−^ mice than in WT. These findings demonstrate multi-phase immune cell changes in remnant kidney after UNx, characterized by rapid innate immune cell activation, gradual adaptive immune cell regulation, and shifts in immune checkpoint levels. These immune changes could influence compensatory changes, including GFR in live kidney donors and others after UNx.

## Introduction

Living donor kidney transplants accounted for 23% of all transplants performed in the United States in 2021^1^. Within 6 months of unilateral nephrectomy (UNx), kidney function measured by glomerular filtration rate (GFR) in donors typically rises to approximately 70% of preoperative values, a phenomenon accompanied by increased kidney size and mass attributed to compensatory renal hypertrophy (CRH) and hyperplasia ^2–4^. CRH following UNx has been extensively studied in clinical and animal models given importance for live donors, transplant recipients as well as patients with native kidney disease with progressive CKD. Structural analyses in rodent UNx models have shown that increases in kidney mass result from hypertrophy of glomerular and proximal tubular cells, as well as hypertrophy and hyperplasia of cortical collecting duct cells ^5–8^ Many physiological changes following UNx may trigger kidney enlargement through different signaling pathways, several of which are also involved in immune cell regulation, suggesting a potential immune cell involvement in CRH. Increased contralateral luminal flow in renal proximal tubules induces rapid changes in fluid shear stress pressure (FSS) ^9,10^, which mechanically signals secretion of autocrine transforming growth factor-β (TGF-β) in epithelial cells of proximal tubules, promoting proliferation and morphological changes in these cells ^11,12^. Previous studies have shown that the mammalian target of rapamycin (mTOR) signaling is also involved in the hypertrophic progression in renal tubules, and deletion of mTOR-S6K1 signaling inhibits renal hypertrophy after nephrectomy ^13,14^. Applying unbiased multi-omics on whole kidney to discover potential signaling mechanisms has shown that the lipid-regulated transcription factor peroxisome proliferator-activated receptor alpha (PPARα) is a determinant of kidney size and is upregulated following nephrectomy^7^. A recent study examined gene expression changes specific to cell types following nephrectomy, identifying FOXM1 and E2F4 as key regulators of the cell cycle and cholesterol biosynthesis. SREBP2, potentially mediated by mTORC1, drives compensatory kidney growth and adaptations to metabolic changes^8^.

While previous studies have to focused on hemodynamic changes or renal tubular epithelial responses, there have been limited studies examining immune responses in the remnant kidney during CRH. In this study, we explored changes in immune cells during CRH after UNx in mice using advanced spectral flow cytometry and single-cell RNA sequencing (scRNA-seq), comparing them to kidney weights and direct GFR measurements. In addition, to begin studying cause and effect, we evaluated kidney changes in UNx Rag1−/− mice, which lack T and B cells.

## Results

### Weight and GFR of the remnant kidneys after UNx

**Figure 1:**
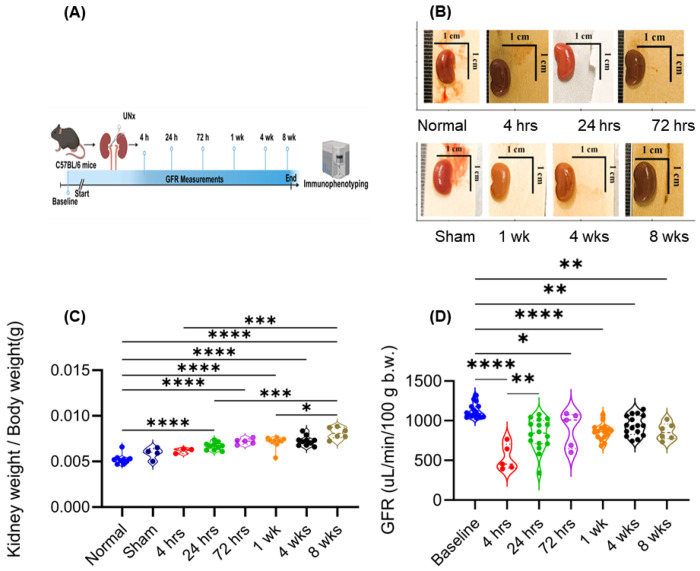
Size and glomerular filtration rate changes in remnant kidney following uninephrectomy in mice. **(A)** Schematic representation of experimental design. (**B)** Representative images depict the size of the remaining kidney at sacrifice throughout the 8-week recovery period of nephrectomized WT mice. **(C)** Kidney weight-to-body weight ratios were calculated at the time of sacrifice: 4 hours (n = 3), 24 hours (n = 10), 72 hours (n = 5), 1 week (n = 10), 4 weeks (n = 10), and 8 weeks (n = 6) after UNx, as well as for normal mice (n = 14). **(D)** Short- and long-term partial restoration of kidney function in WT mice was observed during the 8-week period following surgery. GFR measurements were collected at baseline (n = 16), 4 hours (n = 5), 24 hours (n = 16), 72 hours (n = 5), 1 week (n = 16), 4 weeks (n = 16), and 8 weeks (n = 6). Data are presented as mean ± SEM and analyzed using a one-way ANOVA with Tukey’s post hoc analysis. UNx, unilateral nephrectomy.

We measured changes in remnant kidney size at 4, 24, and 72 hours, as well as at 1, 4, and 8 weeks after left kidney UNx (Fig. 1B, C). In the early period after nephrectomy, changes to kidney weight-to-body weight ratio (KW: BW) were found at 24 hours after nephrectomy (5.45 ± 0.15g/g at baseline versus 6.68 ± 0.12 mg/g at 24 hours; P < 0.0001). KW: BW at 8 weeks (8.11 ± 0.25 mg/g) was greater than at 4 hours (6.17 ± 0.16 mg/g; P < 0.0001), 24 hours (6.68 ± 0.12; P < 0.0001), and 1 week (7.11 ± 0.20 mg/g; P < 0.05).

Compared to baseline (1124 ± 22.48 μL/min/100g b.w.), GFR sharply dropped at 4 hours after UNx (534.7 ± 72.89 μL/min/100g b.w., 47.56 ± 6.483% at 4 hr; P < 0.0001) (Fig. 1D). At 24 hours, GFR increased from its initial drop at 4 hours to 828.2 ± 49.77 μL/min/100g b.w. (73.51 ± 4.43% average baseline; P < 0.05), maintaining throughout the remaining measured recovery period (890.8 ± 101.5 μL/min/100g b.w., 79.23 ± 9.02% at 72 hours; 860.2 ± 25.28 μL/min/100g b.w., 76.30 ± 2.21 at 1 week; 940.4 ± 31.80 μL/min/100g b.w., 83.44 ± 2.84% at 4 weeks; 862.9 ± 43.64 μL/min/0g b.w., 76.75 ± 3.88% at 8 weeks).

### Kidney immune cells change quantitatively after UNx.

**Figure 2:**
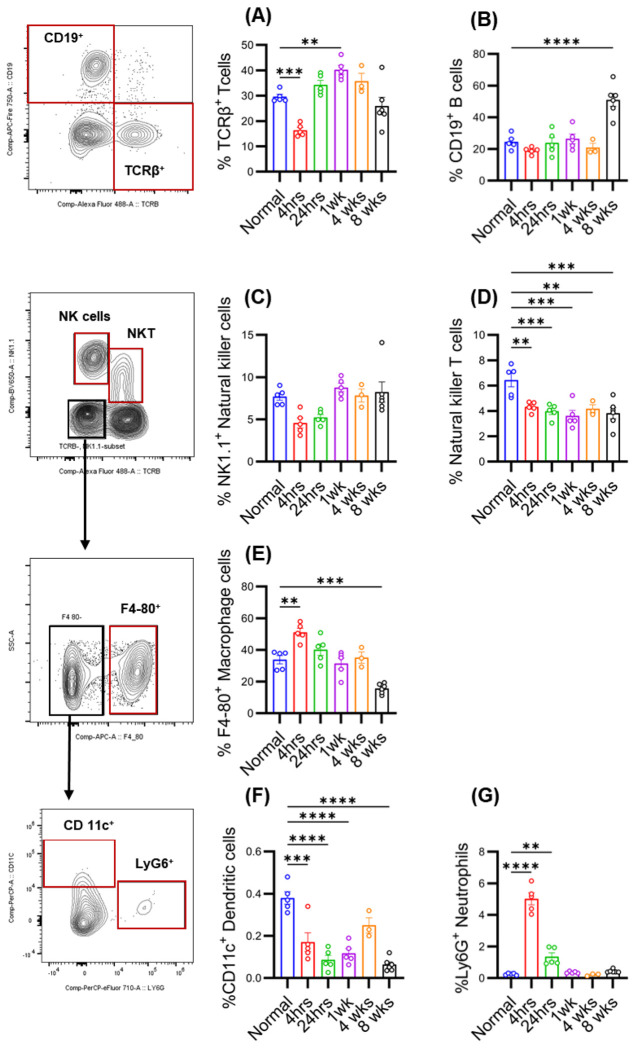
Uninephrectomy changes immune cell populations in the remnant kidney. **(A)** TCRβ^+^ T cells**, (B)** B cells**, (C)** NK cells**, (D)** NKT cells, **(E)** Macrophages, (**F)** Dendritic cells, and **(G)** Neutrophils, change as a percentage of CD45+ immune cells after UNx. Data are presented as mean ± SEM. N=5-6 per group (except 4 weeks group n=3), with each dot representing an individual mouse. Statistical comparisons were performed using one-way ANOVA with Tukey’s post hoc analysis. UNx, unilateral nephrectomy.

The effects of UNx on immune cell populations in the remnant kidney were studied using flow cytometry. For T cell subsets, B cells, and other immune cells, we gated on the total CD45 population using the gating strategies outlined in Fig. 1. We measured both percentage and absolute number changes. TCRβ^+^ T cells show a significant decrease at 4 hours compared to the normal “control” (16.38 ± 1.06% versus 29.60 ± 0.99%; p < 0.001). By 1 Week, the T cell population increases compared to the normal control (40.24 ± 1.75% vs. 29.60 ± 0.99%; p < 0.01). Furthermore, T cells remain stable over the 8-week period, with a declining trend after UNx (Fig. 2A). The CD19^+^ B cell population, we noted a significant increase at 8 weeks as a long-term immune cell population change (Fig. 2B) compared to the normal control (42.85 ± 4.74% vs. 22.66%±2.08; *p* < 0.001).

The NK cell population decreased early after UNx compared to the normal control, with the percentage of NK cells reaching its lowest point between 4 and 24 hours (4.5 ± 0.58%; 5.21 ± 0.3 versus 7.71 ± 0.43% p = 0.0700), though this difference was not statistically significant. (Fig. 2C). However, the NK cell population later recovered after UNx, with their percentages at later time points (8.77 ± 0.50% at 1 week, 7.82 ± 1.58% at 4 weeks, and 8.24 ± 1.20% at 8 weeks) becoming comparable to the control (7.71 ± 0.43%) (Fig. 2C). A decrease in the NKT cell population was noted at nearly all the time points 4hours (4.32 ± 0.52%; *p* < 0.05), 24hours (3.97 ± 52%; *p* < 0.01), 1week (3.60 ± .52%; p < 0.0001), 4weeks (4.17 ± 0.60%; p < 0.0001) and 8 weeks (3.82 ± 0.49%; p < 0.0001) compared to normal group (6.44 ± 0.54%) (Fig. 2D).

We observed an increase in F4/80^+^ macrophages at 4 hours after UNx compared with the normal control group (51.18 ± 2.52% vs. 33.84 ± 2.61%; *p* < 0.05). However, the percentage of macrophage cells at 8 weeks was significantly decreased (15.68 ± 1.26%; *p* < 0.01) (Fig. 2E). Dendritic cells (CD11c^+^) decreased at 4 hours (0.17 ± 0.04% vs. 0.38 ± 0.03%; *p* < 0.0001), 24 hours (0.09 ± 0.02% vs. 0.38 ± 0.03%; *p* < 0.0001), and 1week compared to baseline (0.11 ± 0.02% vs. 0.38 ± 0.03%; *p* < 0.0001) and 8 weeks (0.063 ± 0.01% vs. 0.38 ± 0.03%; *p* < 0.0001) (Fig. 2F). We also noted an increase in neutrophils (Ly6G^+^) compared to the normal control (0.25 ± 0.02%) at early time points, specifically at 4 hours (5.02 ± 0.37%; *p* < 0.0001) and at 24 hours (1.36 ± 0.24%; *p* < 0.01) (Fig. 2G).

### Kidney immune T cells undergo quantitative and phenotypic changes after UNx.

**Figure 3:**
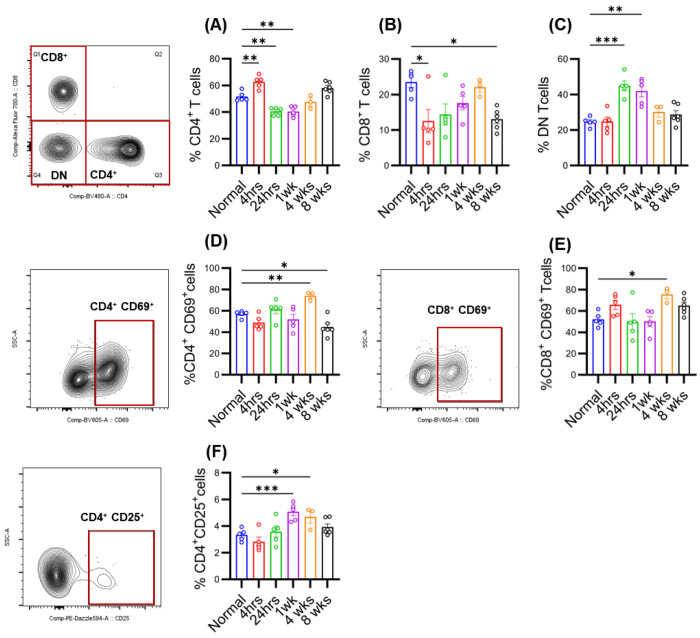
Uninephrectomy increases Double Negative T cells in the remnant kidney, with a gradual decrease over time. **(A)** CD4^+^ cells, **(B)** CD8^+^ cells, and **C)** DN (TCRβ^+^CD4^−^CD8^−^) cells. T cell activation Markers (**D)** CD69^+^CD4^+^, (**E)** CD69+CD8+, and (**F**) CD25+CD4+. Data are presented as mean ± SEM. N=5-6 per group (except 4 weeks group n=3), with each dot representing an individual mouse. Statistical comparisons were conducted using one-way ANOVA with Tukey’s post hoc analysis.

In T cell populations, we observed a significant increase in CD4 + T cells at 4 hours (Fig. 3A) compared to the normal control (62.68 ± 2.1% versus 51.60 ± 1.6%; p < 0.01). The CD4 + T cell population decreased at 24 hours (40.50 ± 1.3% vs. 51.60 ± 1.6%; p < 0.01) and after 1 week compared to the normal control (40.30 ± 1.7% vs. 51.60 ± 1.6%; p < 0.01). However, CD4 + T cells recover over 8 weeks after UNx (Fig. 3A) compared to the normal control group.

The percentages of CD8 + T cells decreased significantly at 4 hours (12.50 ± 3.2%; p < 0.05) (Fig. 3B). The CD8 + T cell population recovered to normal levels over time at 1 week (17.60 ± 1.8%) and 4 weeks (22.07 ± 1.3%), returning to control baseline levels (23.52 ± 1.4%) (Fig. 3B). CD8 + T cells decreased again at 8 weeks after UNx (13.03 ± 1.8%; p < 0.05). TCRβ + CD4^−^ CD8^−^ double-negative (DN) T cells are recognized as anti-inflammatory T cells ^15,16^. DN T cells significantly increased at 24 hours (44.94 ± 2.7% vs. 24.60 ± 1.1%; p < 0.001) and 1 week (41.90 ± 2.73% vs. 24.60 ± 1.1%; p < 0.01) after UNx compared to the control group (Fig. 3C).

Compared to normal controls, CD69 + CD4+ T resident cells increased significantly at 4 weeks (74.10 ± 2.40% vs. 57.04 ± 1.42%, p < 0.01) and decreased at 8 weeks (44.85 ± 3.3% vs. 57.04 ± 1.427%, p < 0.05) (Fig. 3D). Similarly, CD69 + CD8+ T resident cells increased significantly at 4 weeks (75.53 ± 4.1% vs. 52.02 ± 2.9%, p < 0.05) compared to normal controls (Fig. 3E). CD4 + CD25+ T cells showed an increasing trend over time, with significant increases at 1 week and 4 weeks compared to normal controls (5.06 ± 0.29% vs. 3.3 ± 0.20%, p < 0.05, and 4.6 ± 0.48% vs. 3.3 ± 0.20%, p < 0.05, respectively) (Fig. 3F).

**Figure 4:**
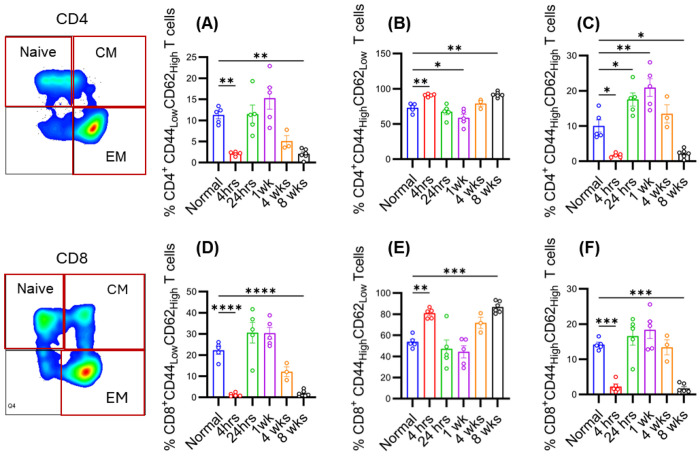
Memory phenotype markers for naïve T cells (CD44^−^CD62L^+^), effector memory T cells (CD44^+^CD62L^−^), and central memory T cells (CD44^+^CD62L^+^) after UNx. Panels (**A, B,** and **C)** demonstrate data for CD4 cells, while panels (**D, E,** and **F)** show data for CD8 cells. Data are presented as mean ± SEM, with each dot representing an individual mouse. Sample sizes are 5-6 per group, except for the 4-week group (n=3), with each dot representing an individual mouse. Statistical comparisons were conducted using one-way ANOVA with Tukey’s post hoc test.

We used phenotypic markers CD44 and CD62L in flow cytometry to assess the activation status of different T cell subtypes. Compared to the normal control, the percentage of naïve (CD44 low, CD62L high) CD4 + T cells showed a fluctuating trend and decreased at 4 hours (2.10 ± 2.0% vs. 11.26 ± 0.86%; p < 0.01) during the early acute injury phase. However, it recovered during the intermediate phase (24 hours to 1 week) and then gradually decreased over time up to 8 weeks (2.05 ± 0.47% vs. 11.26 ± 0.86%; p < 0.01) (Fig. 4A). Effector memory (CD44 high, CD62L low) CD4 + T cells significantly decreased at 1 week (58.66 ± 5.67% vs. 73.02 ± 2.8%, p < 0.05) after an initial increase at 4 hours (90.90 ± 0.8% vs. 73.02 ± 2.8%, p < 0.01) and later at 8 weeks (91.75 ± 1.69% vs. 73.02 ± 2.8%, p < 0.01) (Fig. 4B). The central memory phenotype (CD44 high, CD62L high) cells significantly decreased at both 4 hours (1.69 ± 0.2% vs. 10.02 ± 1.7%; p < 0.01) and 8 weeks (2.1 ± 0.49% vs. 10.02 ± 1.7%; p < 0.01). However, it increased during the intermediate phases, i.e., at 24 hours (17.5 ± 1.8% vs. 10.02 ± 1.7%; p < 0.01) and 1 week (20.9 ± 2.5% vs. 10.02 ± 1.7%; p < 0.01) after UNx, compared with the control group (Fig. 4C).

Similarly, in the CD8 T cell subset, the percentage of naïve CD8 + T cells followed a pattern similar to that of CD4+ naïve cells, fluctuating and decreasing sharply at 4 hours (1.23 ± 0.36% vs. 22.20 ± 1.80%, p < 0.0001) and at 8 weeks after UNx (1.8 ± 0.56% vs. 22.20 ± 1.80%, p < 0.0001) (Fig. 4D). Effector memory CD8 + T cells increased significantly at 4 hours (80.90 ± 2.1% vs. 53.70 ± 2%; p < 0.01) and at 8 weeks compared to the normal group (86.73 ± 2.20% vs. 53.70 ± 2.5%; p < 0.001) (Fig. 4D). The central memory phenotype cells decreased significantly at 4 hours (2.25 ± 0.74% vs. 14.16 ± 0.6%; p < 0.001) and later at 8 weeks (1.71 ± 0.51% vs. 14.16 ± 0.6%; p < 0.001). However, recovered during the intermediate phases compared to the control baseline (14.16 ± 0.6%) after UNx (Fig. 4F).

**Figure 5:**
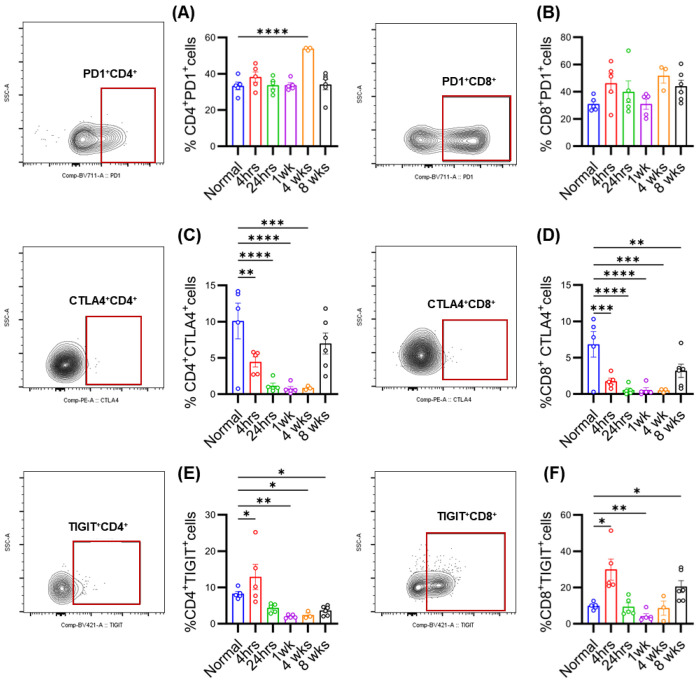
Immune checkpoint inhibitors CTLA4 and TIGIT decreased post UNx in the remnant kidney. No change in Immune checkpoint inhibitor PD-1 in both the CD4 and CD8 subsets, except for a 4-week increase in CD4. **(A)** PD-1 CD4+ cells; **(B)** PD-1 CD8^+^ cells. CTLA4 decreased in both CD4 **(C)** and CD8 **(D)** except at 8 weeks. TIGIT+ CD4 **(E)** and CD8 **(F)** decreased except for 4 hours of immediate surgical response to injury. Data are presented as mean ± SEM. N=5-6 per group (except 4-week group n=3), with each dot representing an individual mouse. Statistical comparisons were conducted using one-way ANOVA with Tukey’s post hoc analysis.

We analyzed immune checkpoint molecules, including programmed cell death protein 1 (PD-1), cytotoxic T-lymphocyte-associated protein 4 (CTLA-4), and T cell immunoreceptor with Ig and immunoreceptor tyrosine-based inhibitory motif (ITIM) domains (TIGIT), across various T cell subsets. Compared to the normal group, the percentages of CD4 + PD-1 + T cells were significantly higher at 4 weeks (53.83 ± 0.46% vs. 33.34 ± 2.156%, p < 0.05) (Fig. 4A). However, there was no change in CD8 + PD-1 + cells compared to normal control after UNx (Fig. 5B). A decrease in the CTLA-4 cell population was observed at nearly all time points in CD4 cells: 4 hours (4.4 ± 0.70%; p < 0.05), 24 hours (1.15 ± 0.37%; p < 0.01), 1 week (0.74 ± 0.30%; p < 0.0001), and 4 weeks (0.85 ± 0.15%; p < 0.0001), except at 8 weeks compared to the normal group (10.09 ± 2.456%) (Fig. 5C). A similar trend was seen in CD8 cells at 4 hours (1.7 ± 0.41%; p < 0.05), 24 hours (0.55 ± 0.29%; p < 0.01), 1 week (0.54 ± 0.33%; p < 0.0001), and 4 weeks (0.50 ± 0.14%; p < 0.0001), with the exception of 8 weeks compared to the normal group (6.83 ± 1.7%) (Fig. 5D). Additionally, there was an increase in TIGIT expression on CD4 cells at 4 hours compared to the normal control (12.8 ± 3.4% vs. 8.24 ± 0.61%, p < 0.05) (Fig. 4E). However, it decreased at 1 week (1.98 ± 0.47%; p < 0.01), 4 weeks (2.38 ± 0.56%; p < 0.05), and 8 weeks (3.65 ± 0.518%; p < 0.05) compared to the normal group (8.24 ± 0.61%) (Fig. 5E). A similar trend was observed in CD8 cells at 4 hours compared to the normal control (29.94 ± 5.8% vs. 9.88 ± 0.84%, p < 0.05) and 8 weeks (20.47 ± 3.29% vs. 9.88 ± 0.84%, p < 0.05) (Fig. 4E). However, their levels decreased at 1 week (4.240 ± 1.279%; p < 0.01), relative to the normal group (9.88 ± 0.84%) (Fig. 5F). Absolute cell counts support the immunophenotyping data; results are shown in Suppl. Fig. S1–3

### scRNAseq analysis of immune cells after UNx

**Figure 6:**
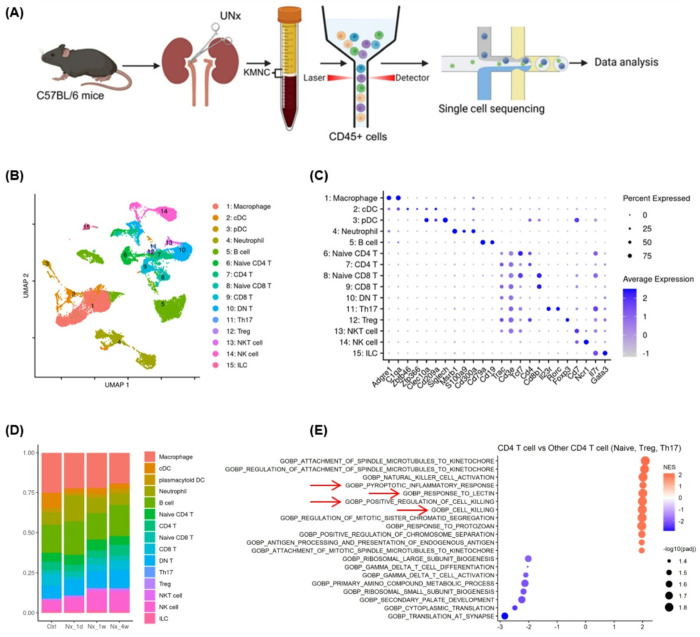
Single-cell transcriptome profile of immune cells in the remnant kidney after uninephrectomy. **(A)** Schematic experimental design. Kidney mononuclear cells were isolated from tissue and stained with an anti-CD45 APC-Cy7 monoclonal antibody for further flow sorting of CD45+ leukocytes before scRNA-seq. **(B)** Uniform Manifold Approximation and Projection (UMAP) showing the annotated cell population. **(C)** Dot plot showing the expression of cell–type–specific marker genes. The size of dots indicates the proportion of cells within a cell population that express a given gene. The color represented the average expression of each gene. **(D)** Bar plots showing the cell ratio of immune cell subsets in each time point. **(E)**Dot Plot showing GSEA analysis of CD4 T cell vs other CD4 T cell including Naïve CD4 T, Treg, and Th17 populations.

To elucidate the characteristics of the immune cells after nephrectomy, we performed scRNA-seq of CD45 + cells isolated from Control (Normal) and the remnant kidney at 1 day, 1 and 4 weeks after nephrectomy. (Fig. 6A). After quality control, we obtained 24,958 single-cell transcriptomes. After dimension reduction and clustering, scRNA-seq data were classified into 15 clusters based on known marker genes (Fig. 6B, C). Cell clusters included Macrophage (Adgre1, C1qa), classical dendritic cell (cDC) (Zbtb46, Zfp366, Clec10a, Cd209a), plasmacytoid DC (pDC) (Siglech), Neutrophil (Msrb1, S100a9, Cd300a), B cell (Cd79a, Cd19), Naïve CD4 T (Trac, Cd3e, Cd4, Tcf7), CD4 T (Trac, Cd3e, Cd4), Naïve CD8 T (Trac, Cd3e, Cd8b1, Tcf7), CD8 T (Trac, Cd3e, Cd8b1), DN T (Trac, Cd3e), Treg (Trac, Cd3e, Cd4, Foxp3+), Th17 (Trac, Cd3e, Cd4, Il23r, Rorc), NKT cell (Trac, Cd3e, Cd7), NK cell (Cd7, Ncr1), ILC (Il7r, Gata3). CD4 T was increased, while B cell, Naïve CD4 T, Naïve CD8 T, CD8 T, and DN T were unchanged or decreased (Fig. 6D). GSEA analysis revealed that CD4 T cell-enriched genes involved in NATURAL_KILLER_CELL_ACTIVATION (Zfp683, Il21, Nkg7, Slamf7, Klre1, Cd160, etc), PYROPTOTIC_INFLAMMATORY_RESPONSE (Casp1, Gzmb, Gzma, Casp4, Trem2, Naip2, etc), POSITIVE_REGULATION_OF_CELL_KILLING (Klrc1, Xcl1, Il21, Ifng, Klrc2, Ccr5, etc) and ANTIGEN_PROCESSING_AND_PRESENTATION_OF_ENDOGENOUS_ANTIGEN (H2-Q6, H2-Q10, H60b, H2-M2, H2-Q7, Ulbp1, etc) (Fig. 6E).

### Effects of T and B cell deficiency on kidney weight and GFR after UNx

**Figure 7:**
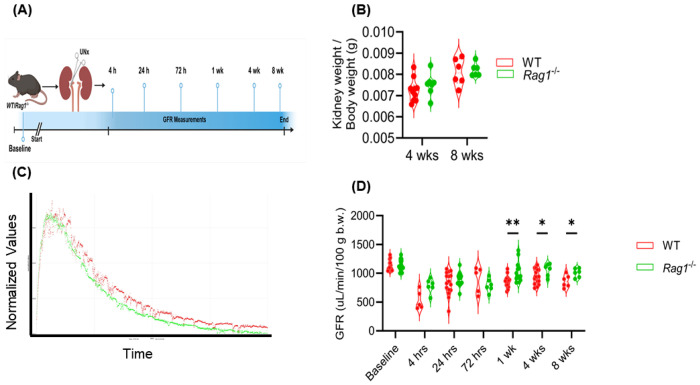
*Rag1*−/− mice had increased glomerular filtration rate in the remnant kidney after uninephrectomy compared to wild-type mice. **(A)** Schematic representation of the experimental design of UNx in *Rag1*−/− vs. WT. Sham mice underwent anesthesia and skin and muscle incision, but had no kidney tissue removed. **(B)** Kidney weight-to-body weight comparisons between WT and *Rag1*^−/−^ mice at 4-week (WT n = 10; *Rag1*−/− n = 7) and 8-week (WT n = 6; *Rag1*−/− n = 6) timepoints. **(C)** A comparison of normalized GFR values between WT and Rag1−/− mice (8 weeks) indicates that sinestrin is cleared more rapidly over time in Rag1−/− mice. **(D)** Comparison of kidney functional recovery for WT and *Rag1*^−/−^ mice following UNx at baseline (WT n = 16; *Rag1*−/− n = 14), 4 hours (WT n = 5; *Rag1*−/− n = 6), 24 hours (WT n = 16; *Rag1*−/− n = 13), 72 hours (WT n = 5; *Rag1*−/− n = 6), 1 week (WT n = 16; *Rag1*−/− n = 13), 4 weeks (WT n = 16; *Rag1*−/− n = 6), and 8 weeks (WT n = 6; *Rag1*−/− n = 6). Data are presented as mean ± SEM and analyzed using unpaired nonparametric Mann-Whitney tests. GFR, glomerular filtration rate; UNx, unilateral nephrectomy.

To begin to investigate the cause-and-effect role of immune cells on CRH, we studied UNx in Rag1−/− mice, which lack mature T and B cells (Fig. 7A). There was no difference between KW: BW of Rag1−/− and WT mice at 4 weeks (7.51 ± 0.20 versus 7.26 ± 0.17 mg/g; p > 0.05) and 8 weeks (8.21 ± 0.12 versus 8.11 ± 0.25 mg/g; p > 0.05) after UNx (Fig. 7B).

Similar to WT, Rag1−/− mice had an initial drop in GFR following UNx (1129 ± 26.9 μL/min/100g b.w. at baseline versus 783.4 ± 49.59 μL/min/100g b.w. at 4 hours). However, Rag1−/− GFR was increased compared to WT at 1 week (1024 ± 49.05 versus 860.2 ± 25.28 μL/min/100g b.w.; p < 0.01), 4 weeks (1062 ± 44.49 versus 940.4 ± 31.80 μL/min/100g b.w.; p < 0.05), and 8 weeks (1008 ± 32.02 versus 862.9 ± 43.64 μL/min/100g b.w.; p < 0.05) after UNx (Fig. 7C, D).

## Discussion

We performed detailed quantitative and transcriptional analysis of kidney immune cells during compensatory renal hypertrophy and uninephrectomy in mice. The goal was to produce an immune cell map of the remnant kidney simulating a live kidney donor, and this hypothesis generating data would serve as a basis for future mechanistic studies. We found a well-coordinated, time-sensitive immune response that begins with a rapid innate cell phase that transitions into regulated adaptive immune cell responses. The UNx timeline can be divided into three phases: acute injury (0–24 hours), recovery (24 hours to 1 week), and immune remodeling (4–8 weeks). During acute injury, innate immune cells are engaged with rapid infiltration of neutrophils and macrophages, accompanied by a decrease in NK cells. The proportion of total T cells (TCRβ) decreased at 4 hours and returned to baseline at 24 hours. This transient relative decrease is likely due to an immediate influx of innate immune cells following surgical intervention, as confirmed by absolute cell number data. NKT cells and dendritic cells declined steadily throughout the study period. In the recovery phase, innate immune cell percentages returned to baseline, while T cell percentages increased. During immune remodeling, T cells, macrophages, and dendritic cells decreased, whereas B cells increased. Quantitative and molecular changes in immune cells provided an immunologic map of temporal changes following nephrectomy. Preliminary, hypothesis-testing studies in mice deficient in T and B cells demonstrated an increase in GFR at select time points compared to WT mice.

In the kidney, activated pro-inflammatory lymphocytes can damage tissue and contribute to injury processes, while anti-inflammatory lymphocytes play protective roles ^16,17^. The balance and interactions of these lymphocyte subsets may influence the long-term function of the remaining kidney. The initial response to UNx alters T cell subsets: during acute injury, CD4 T cells increased at 4 hours, while CD8 T cells decreased significantly, and TCRαβ^+^ CD4^−^ CD8^−^ (DN) T cells remained unchanged. During recovery, both CD4 and CD8 T cells decreased, whereas DN T cells increase significantly between 24 hours and one week. DN T cells are considered anti-inflammatory and typically constitute up to 20% of TCRαβ^+^ T cells in healthy mouse kidneys ^18^.

Following nephrectomy, naïve T-cell frequencies decreased, accompanied by a temporary increase in effector-memory cells during acute injury. Between 24 hours and 1 week, central-memory T cells increased, resulting in a balanced pool of central and effector-memory cells by 4–8 weeks. The decline in naïve cells alongside a rise in effector memory cells may reflect adaptive remodeling in the remnant kidney ^19^. Naïve T cells primarily circulate between the blood and lymphoid organs and become activated after injury. Central memory T cells possess greater proliferative capacity and are home to lymphoid tissues, whereas effector memory T cells rapidly migrate to inflamed tissues and initiate inflammation ^20^

Immune cell activation is a key mechanism enabling detection and response to damaged cells. The proportion of CD69^+^ T cells, an early activation marker essential for lymphocyte migration and proliferation ^21,22^, increased significantly on both CD4^+^ and CD8^+^ cells during the 4-week period. Additionally, CD4^+^CD25^+^ cells increased over time after UNx. Although most regulatory T cells (Tregs) express CD25, only a subset constitutes the Treg population. ^20,23^.

Programmed cell death protein-1 (PD-1), cytotoxic T-lymphocyte antigen 4 (CTLA-4), and T-cell immunoglobulin and ITIM domain (TIGIT) are major immune co-inhibitory receptors ^24^. PD1 expression increased significantly by 4 weeks, particularly in CD4^+^ T cells, with approximately 50% expressing PD1 at levels markedly higher than in normal groups. CTLA-4 and TIGIT decreased across CD4^+^ and CD8^+^ T cells, followed by a gradual increase at 8 weeks, indicating diminished checkpoint control during recovery. High PD1 expression is often associated with prolonged antigen exposure and T cell exhaustion^25,26^. These findings suggest that chronic inflammation in the remnant kidney may eventually lead to T cell exhaustion, following an initial stress-related decrease in PD1 expression.

Single-cell data analyzed using GSEA revealed that CD4 T cell-enriched genes involved in natural killer cell activation, pyroptotic inflammatory response, positive regulation of cell killing, and antigen processing and presentation of endogenous antigens during the late immune cell remodeling phase were consistent with flow cytometry data. However, it is unclear why there was in increase in B cells during the 8-week immune remodeling phase.

Innate immune cells exhibited significant changes during acute injury (0–24 hours): neutrophils and macrophages increased, whereas dendritic cells, NK cells, and NKT cells decreased. Surgical intervention often induces abrupt changes in innate immune cells, particularly during acute injury ^27^. Neutrophils and macrophages returned to normal levels, except that macrophage contued to be decreased at 8 weeks. Macrophages play a critical role in responding to tissue damage and are versatile cells that transition from the M1 pro-inflammatory state to the M2 pro-regenerative state once injury has been resolved.^28^ Additionally, macrophages facilitate tissue repair by releasing chemokines that recruit immune and progenitor cells to the affected area, thereby enhancing the regenerative capacity of tubular epithelial cells^28,29^.

Persistent decreases in dendritic cells and NKT cells were noted at all time points post-UNx. Dendritic cells, highly specialized antigen-presenting cells derived from the bone marrow, play a key role in triggering, coordinating, and regulating both innate and adaptive immune responses. They respond to damage-associated molecular patterns (DAMPs) through pattern recognition receptors, thereby driving inflammation and initiating immune defenses^30^. Notably, unlike ischemia or acute injury models in which dendritic cell populations expand following tissue injury, UNx triggered a sustained decline in renal dendritic cells. Studies have shown that limiting dendritic cell capacity to activate NKT cells or boosting regulatory T cell function can affect immune regulation.^30^ The ongoing decrease in dendritic cells could indicate reduced renal inflammatory signaling after UNx and limited dendritic cell recruitment. Similarly, the long-term decrease in NKT cells may help protect the remnant kidney from NKT-mediated cytotoxic damage, as activated NKT cells are known to cause tubular and endothelial injury in acute kidney injury^31^.

Rag1−/− mice are genetically engineered to lack functional T and B lymphocytes^32^. We initially hypothesized that these mice would have reduced GFR after UNx if these cells contributed to compensatory increases in function. However, improved GFR recovery was observed in Rag1−/− mice after UNx. In WT mice it is possible that immune activation, checkpoint signaling, and ongoing modulation, collectively limiting complete functional recovery. Therefore, T and B cells might play early roles in regulating GFR after UNx.

Our study had several limitations. For example, the UNx model does not precisely replicate human conditions, though we removed the left kidney as most live kidney donors do. We used normal, healthy mice aged 8 to 9 weeks, while most current human live donors are not young and healthy; they may be older or have conditions such as hypertension, obesity, and hyperlipidemia. We performed scRNA-seq on flow-sorted kidney immune cells to enhance our immune cell-focused studies, but we did study the CRH effect on other kidney cells. Notably, our descriptive studies do not establish a causal link between immune cells and CRH. However, we began to examine mechanistic effects using RAG1−/− mice. We believe that the focus on immune cells in CRH after UNx has been relatively understudied, and the data that we have generated with traditional and newer technologies will lay the groundwork for future mechanistic studies in mice and humans.

In conclusion, this study highlights the complex and dynamic kidney immune responses following UNx, which have significant implications for understanding kidney adaptation and immune regulation in the solitary kidney after live kidney donation. These findings will stimulate further research to understand the role of the immune system adaptation to reduced total nephron mass.

## Materials and Methods

### Mice

Male C57BL6/J (WT, Strain 000664) mice and Rag1−/− (B6.129S7-Rag1tm1Mom/J, Strain 002216) mice on BL/6 background lacking mature T and B cells were purchased from the Jackson Laboratory (Bar Harbor, ME) and housed under specific pathogen-free conditions in the Johns Hopkins University animal facility. All mice were 8–9 weeks old at the time of nephrectomy. Animals were euthanized with an i.p. injection of ketamine (200 mg/kg) and xylazine (15 mg/kg) unless otherwise stated. Subsequently, kidney weight was measured. Body weight was recorded prior to GFR assessment. All procedures complied with protocols approved by the Johns Hopkins University Institutional Animal Care and Use Committee (Protocol No. MO24M439).

### Nephrectomy

Mice were anesthetized by intraperitoneal (i.p.) injection of pentobarbital sodium (75 mg/kg) followed by midline laparotomy and removal of the left kidney, often the kidney removed in live donors. While maintaining animal body temperature of 37°C, the renal pedicle and ureter were bluntly dissected then tied off twice with 3 – 0 nonabsorbable thread to reduce chances of breakage and unintended bleeding. Animals were then hydrated with 500 μL of warm (37°C) saline and sutured with 4 – 0 absorbable thread, staying at a constant body temperature until regaining consciousness. Animals were then allowed to recover with ad libitum access to food and water. Sham surgeries consisted of identical processes for anesthesia, skin and muscle incisions, heating, and hydration, but no kidney tissue was removed. Mice in the baseline group did not receive any anesthesia or surgery and were 8 weeks old at the time of sacrifice.

### Assessment of glomerular filtration rate

GFR was directly measured by recording the transcutaneous fluorescein isothiocyanate (FITC)-sinistrin (inulin analogue) with a fluorometer device (Medi-Beacon, St. Louis, MO, USA) as previously described^33^. At baseline, 24 hrs, one week, two weeks, three weeks, and four weeks. The background fluorescence level of the skin was recorded for five minutes, and FITC-sinistrin (0.07 mg/g body weight, Medi-Beacon, St. Louis, MO, USA) was injected via the retro-orbital sinus. After injection, mice were immediately transferred to separate cages to recover from isoflurane anesthesia and remained there for the duration of the measurement period. The fluorometer was programmed to make a transcutaneous measurement every 5 seconds, and measurements were made for 1.5 hrs and stored on the device ^34^. GFR was calculated using a three-compartment model, enabling direct conversion from the elimination half-life, using a published conversion factor.^35^

### Isolation of kidney mononuclear cells

Kidney mononuclear cells (KMNCs) were isolated following an established protocol ^36^. Briefly, mice were anesthetized through an i.p. injection of ketamine (140 mg/kg) and xylazine (10 mg/kg) and exsanguinated to remove circulating immune cells in the kidney. The right kidney was then harvested from each mouse, decapsulated, finely minced, and digested by incubation in collagenase D (5 mg/mL; Sigma-Aldrich) for 30 minutes at 37°C. The digested tissue was mechanically disrupted and filtered through a 70-μm strainer (Corning), then centrifuged at room temperature (3000 RPM for 30 minutes, no acceleration, no brake) in a Percoll density gradient according to the manufacturer’s instructions, after which mononuclear cells were collected. Isolated cells were then resuspended in Roswell Park Memorial Institute (RPMI) media supplemented with 5% fetal bovine serum (FBS) and counted for subsequent antibody staining. After isolation, approximately 1 x 10^6 KMNCs per mouse from five of the mice in each group were designated for flow cytometry, while the remaining KMNCs from all mice were pooled within their experimental groups for cell sorting and downstream scRNA-seq.

### Immunophenotyping

Multiparameter spectral flow cytometric analysis of kidney immune cells was performed according to established protocols^33,37,38^. After KMNC isolation, cells were incubated for 15 minutes at room temperature with anti-CD16/CD32 Fc block (Clone: S17011E, BioLegend). Subsequently, cells were incubated with an antibody cocktail containing fluorochrome-conjugated monoclonal antibodies for 30 minutes. Antibodies were purchased from various sources for reactivity to mouse antigens. The following antibodies were used: CD4-BV480 (Clone: RM4-5), CD8α-AF700 (Clone: 53 – 6.7), CD11c-PerCP (Clone: N418), CD19-APC/Fire 750 (Clone: 6D5), CD25-PE/Dazzle 594 (Clone: PC61), CD44-BV570 (Clone: IM7), CD45-Spark Blue 550 (Clone: 30-F11), CD62L-BV785 (Clone: MEL-14), CD69-BV605 (Clone H1.2F3), CD152-PE (Clone: UC10-4B9), F4/80-APC (Clone: BM8), Ly-6G-PerCP-eFluor 710 (Clone: 1A8-Ly6g), NK1.1-BV650 (Clone: PK136), PD-1-BV711 (Clone: 29F.1A12), TCRβ-AF488 (Clone: H57-597), CTLA-4 (Clone: UC10-4B9)TIGIT-BV421 (Clone: 1G9). Readings were collected using a Cytek Aurora flow cytometer and SpectroFlo acquisition software. Absolute numbers of viable cells were determined using trypan blue exclusion dye and a hemocytometer under light microscopy. The absolute numbers of each cell population in flow cytometry were calculated by multiplying the total KMNC count per kidney by the percentage of each cell population as determined in FlowJo analysis ^39^.

### Kidney CD45 + immune cell sorting

Isolated KMNCs from WT mice (n = 5, each group) were first incubated for 15 minutes at room temperature with anti-CD16/CD32 Fc block (Clone: 2.4G2, BD Biosciences). These cells were stained with the monoclonal antibody anti-mouse CD45 APC-Cy7 (Clone: 30-F11, BioLegend). CD45 + immune cells were sorted using flow cytometry (FACS) with over 80% viability, and propidium iodide (PI) was used to exclude dead cells. Cell sorting was performed using a MoFlo XDP, and purified cells were immediately processed for scRNA-seq analysis.

### Single-cell RNA sequencing

Flow-sorted CD45 + kidney cells were partitioned and barcoded using the 10X Chromium Controller, with approximately 10,000 cells loaded onto the NovaSeq 6000 S4 200-cycle flow cell. The library was constructed using the Chromium Next GEM Single Cell 5′ HT v2 Kit and sequenced on the Illumina NovaSeq 6000 system. The scRNA-seq raw data was aligned to the mm10 reference genome using Cell Ranger (version 7.1.0). Subsequent scRNA-seq analysis was conducted with Seurat (version 5.0.2)^40^. Potential doublets and ambient RNA were removed using SoupX and doubletFinder^41,42^. Low-quality cells were identified and filtered out if their gene counts were < 500 or > 4000, and percentage of mitochondrial gene content were > 10%. Normalization, selection of highly variable genes, and scaling were performed with SCTransform. Before data integration, the datasets were subjected to SelectIntegrationFeatures and PrepSCTIntegration functions with default parameters. The integration anchors were then determined using FindIntegrationAnchors. With these anchors, the function of IntegrateData was used to perform the dataset integration. For the integrated object, PCA was conducted and the top 30 PCs were used for dimension reduction and clustering.Clusters were annotated based on cluster-specific marker genes. Marker genes were identified using the FindMarkers function and only genes with adjusted p values < 0.05 (determined by two-sided Wilcoxon rank-sum test and adjusted using Bonferroni correction) were considered.

### Gene set enrichment analysis

To identify the biological pathway enriched in the corresponding population, we used the R package fgsea (version 1.30.0) with gene ontology biological processes. The permutation number was set to 10,000. The ggplot2 package was used to make figures

### Statistical analysis

Data were expressed as mean ± SEM, indicating the number of animals per group. Comparisons among multiple groups were conducted using one-way or two-way ANOVA, followed by Tukey’s test where appropriate. A single two-group comparison was performed using unpaired nonparametric Mann-Whitney tests. Statistical analysis was conducted using Prism 9 from GraphPad Software, and significance was assessed at P < 0.05.

## Supplementary Material

This is a list of supplementary files associated with this preprint. Click to download.

• SupplFigure.pdf

## Data Availability

All data required to evaluate the conclusions in the paper are present in the paper and the supplementary materials. The scRNA-Seq data from this study have been deposited at the NCBI under the GEO submission (GSE300404).

## Abbreviations

UNx: unilateral nephrectomy
GFR: glomerular filtration rate
CRH: compensatory renal hypertrophy
CKD: chronic kidney disease
scRNA-seq: single-cell RNA sequencing
FSS: fluid shear stress pressure
DN: double-negative
TGF-β: transforming growth factor-β
PPARα: peroxisome proliferator-activated receptor alpha
WT: wild type
KW: BW: kidney weight-to-body weight ratio
Treg: regulatory T cells
